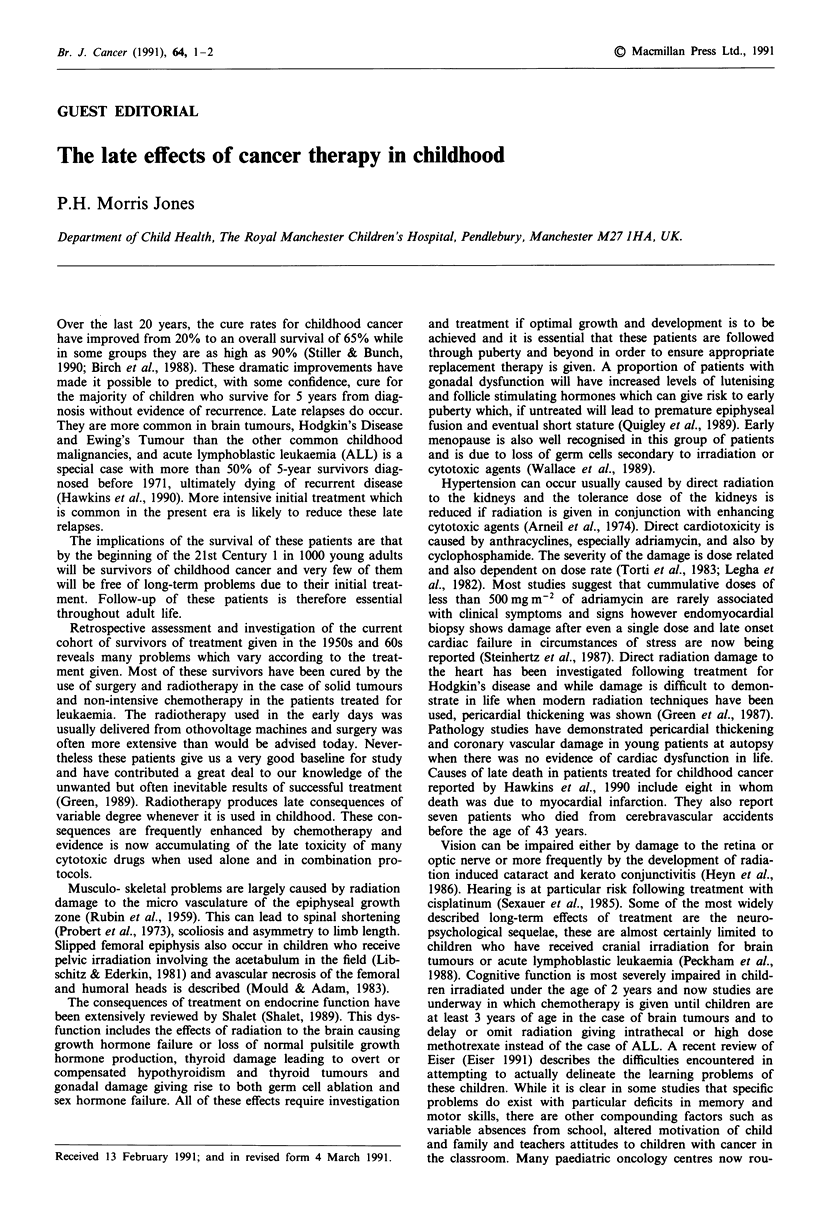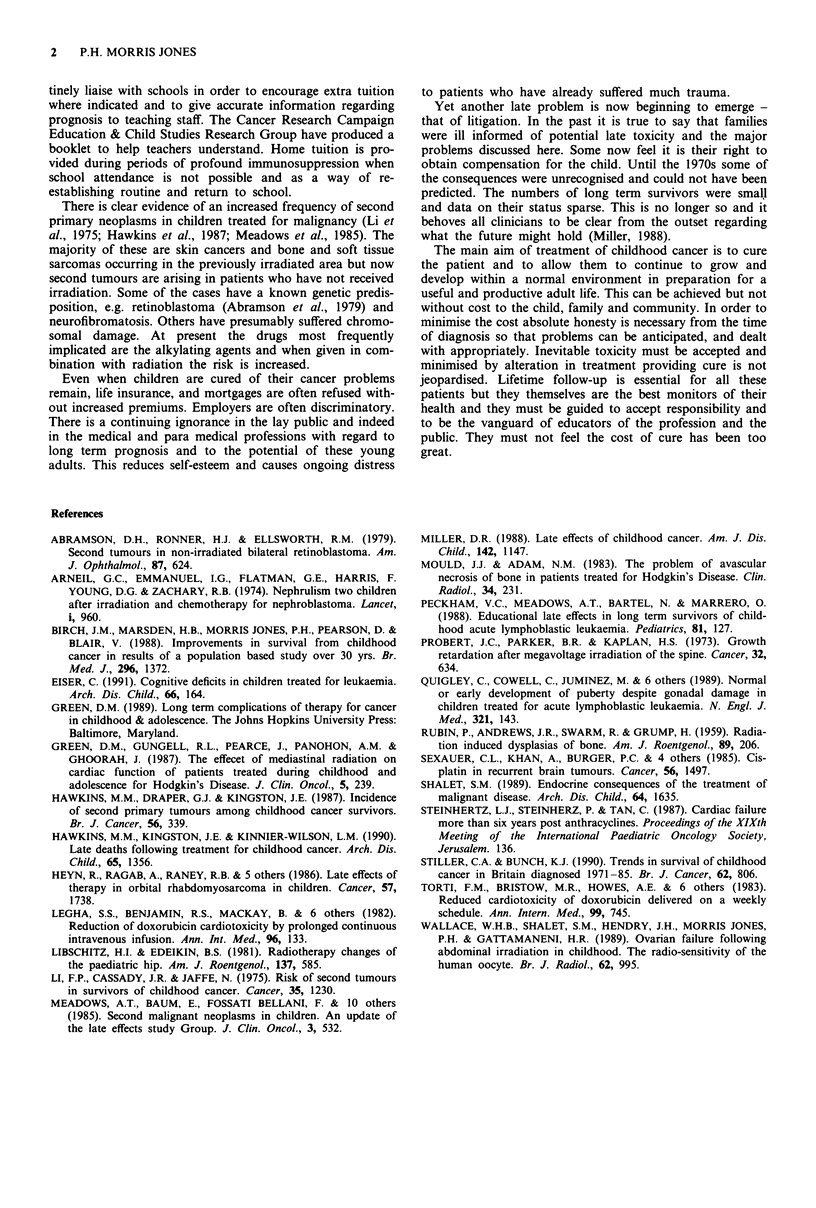# The late effects of cancer therapy in childhood.

**DOI:** 10.1038/bjc.1991.228

**Published:** 1991-07

**Authors:** P. H. Morris Jones


Br. J. Cancer (1991), 64, 1–2 © Macmillan Press Ltd., 1991

GUEST EDITORIAL

### The late effects of cancer therapy in childhood

P.H. Morris Jones

*Department of Child Health, The Royal Manchester Children's Hospital, Pendlebury, Manchester M27 1HA, UK.*

Over the last 20 years, the cure rates for childhood cancer have improved from 20% to an overall survival of 65% while in some groups they are as high as 90% (Stiller & Bunch, 1990; Birch *et al.*, 1988). These dramatic improvements have made it possible to predict, with some confidence, cure for the majority of children who survive for 5 years from diagnosis without evidence of recurrence. Late relapses do occur. They are more common in brain tumours, Hodgkin's Disease and Ewing's Tumour than the other common childhood malignancies, and acute lymphoblastic leukaemia (ALL) is a special case with more than 50% of 5-year survivors diagnosed before 1971, ultimately dying of recurrent disease (Hawkins *et al.*, 1990). More intensive initial treatment which is common in the present era is likely to reduce these late relapses.

The implications of the survival of these patients are that by the beginning of the 21st Century 1 in 1000 young adults will be survivors of childhood cancer and very few of them will be free of long-term problems due to their initial treatment. Follow-up of these patients is therefore essential throughout adult life.

Retrospective assessment and investigation of the current cohort of survivors of treatment given in the 1950s and 60s reveals many problems which vary according to the treatment given. Most of these survivors have been cured by the use of surgery and radiotherapy in the case of solid tumours and non-intensive chemotherapy in the patients treated for leukaemia. The radiotherapy used in the early days was usually delivered from othovoltage machines and surgery was often more extensive than would be advised today. Nevertheless these patients give us a very good baseline for study and have contributed a great deal to our knowledge of the unwanted but often inevitable results of successful treatment (Green, 1989). Radiotherapy produces late consequences of variable degree whenever it is used in childhood. These consequences are frequently enhanced by chemotherapy and evidence is now accumulating of the late toxicity of many cytotoxic drugs when used alone and in combination protocols.

Musculo- skeletal problems are largely caused by radiation damage to the micro vasculature of the epiphyseal growth zone (Rubin *et al.*, 1959). This can lead to spinal shortening (Probert *et al.*, 1973), scoliosis and asymmetry to limb length. Slipped femoral epiphysis also occur in children who receive pelvic irradiation involving the acetabulum in the field (Libschitz & Ederkin, 1981) and avascular necrosis of the femoral and humoral heads is described (Mould & Adam, 1983).

The consequences of treatment on endocrine function have been extensively reviewed by Shalet (Shalet, 1989). This dysfunction includes the effects of radiation to the brain causing growth hormone failure or loss of normal pulsitile growth hormone production, thyroid damage leading to overt or compensated hypothyroidism and thyroid tumours and gonadal damage giving rise to both germ cell ablation and sex hormone failure. All of these effects require investigation and treatment if optimal growth and development is to be achieved and it is essential that these patients are followed through puberty and beyond in order to ensure appropriate replacement therapy is given. A proportion of patients with gonadal dysfunction will have increased levels of lutenising and follicle stimulating hormones which can give risk to early puberty which, if untreated will lead to premature epiphyseal fusion and eventual short stature (Quigley *et al.*, 1989). Early menopause is also well recognised in this group of patients and is due to loss of germ cells secondary to irradiation or cytotoxic agents (Wallace *et al.*, 1989).

Hypertension can occur usually caused by direct radiation to the kidneys and the tolerance dose of the kidneys is reduced if radiation is given in conjunction with enhancing cytotoxic agents (Arneil *et al.*, 1974). Direct cardiotoxicity is caused by anthracyclines, especially adriamycin, and also by cyclophosphamide. The severity of the damage is dose related and also dependent on dose rate (Torti *et al.*, 1983; Legha *et al.*, 1982). Most studies suggest that cummulative doses of less than 500 mg m$^{-2}$ of adriamycin are rarely associated with clinical symptoms and signs however endomyocardial biopsy shows damage after even a single dose and late onset cardiac failure in circumstances of stress are now being reported (Steinhertz *et al.*, 1987). Direct radiation damage to the heart has been investigated following treatment for Hodgkin's disease and while damage is difficult to demonstrate in life when modern radiation techniques have been used, pericardial thickening was shown (Green *et al.*, 1987). Pathology studies have demonstrated pericardial thickening and coronary vascular damage in young patients at autopsy when there was no evidence of cardiac dysfunction in life. Causes of late death in patients treated for childhood cancer reported by Hawkins *et al.*, 1990 include eight in whom death was due to myocardial infarction. They also report seven patients who died from cerebravascular accidents before the age of 43 years.

Vision can be impaired either by damage to the retina or optic nerve or more frequently by the development of radiation induced cataract and kerato conjunctivitis (Heyn *et al.*, 1986). Hearing is at particular risk following treatment with cisplatinum (Sexauer *et al.*, 1985). Some of the most widely described long-term effects of treatment are the neuropsychological sequelae, these are almost certainly limited to children who have received cranial irradiation for brain tumours or acute lymphoblastic leukaemia (Peckham *et al.*, 1988). Cognitive function is most severely impaired in children irradiated under the age of 2 years and now studies are underway in which chemotherapy is given until children are at least 3 years of age in the case of brain tumours and to delay or omit radiation giving intrathecal or high dose methotrexate instead of the case of ALL. A recent review of Eiser (Eiser 1991) describes the difficulties encountered in attempting to actually delineate the learning problems of these children. While it is clear in some studies that specific problems do exist with particular deficits in memory and motor skills, there are other compounding factors such as variable absences from school, altered motivation of child and family and teachers attitudes to children with cancer in the classroom. Many paediatric oncology centres now rou-

Received 13 February 1991; and in revised form 4 March 1991.

tinely liaise with schools in order to encourage extra tuition where indicated and to give accurate information regarding prognosis to teaching staff. The Cancer Research Campaign Education & Child Studies Research Group have produced a booklet to help teachers understand. Home tuition is provided during periods of profound immunosuppression when school attendance is not possible and as a way of re-establishing routine and return to school.

There is clear evidence of an increased frequency of second primary neoplasms in children treated for malignancy (Li *et al.*, 1975; Hawkins *et al.*, 1987; Meadows *et al.*, 1985). The majority of these are skin cancers and bone and soft tissue sarcomas occurring in the previously irradiated area but now second tumours are arising in patients who have not received irradiation. Some of the cases have a known genetic predisposition, e.g. retinoblastoma (Abramson *et al.*, 1979) and neurofibromatosis. Others have presumably suffered chromosomal damage. At present the drugs most frequently implicated are the alkylating agents and when given in combination with radiation the risk is increased.

Even when children are cured of their cancer problems remain, life insurance, and mortgages are often refused without increased premiums. Employers are often discriminatory. There is a continuing ignorance in the lay public and indeed in the medical and para medical professions with regard to long term prognosis and to the potential of these young adults. This reduces self-esteem and causes ongoing distress to patients who have already suffered much trauma.

Yet another late problem is now beginning to emerge – that of litigation. In the past it is true to say that families were ill informed of potential late toxicity and the major problems discussed here. Some now feel it is their right to obtain compensation for the child. Until the 1970s some of the consequences were unrecognised and could not have been predicted. The numbers of long term survivors were small and data on their status sparse. This is no longer so and it behoves all clinicians to be clear from the outset regarding what the future might hold (Miller, 1988).

The main aim of treatment of childhood cancer is to cure the patient and to allow them to continue to grow and develop within a normal environment in preparation for a useful and productive adult life. This can be achieved but not without cost to the child, family and community. In order to minimise the cost absolute honesty is necessary from the time of diagnosis so that problems can be anticipated, and dealt with appropriately. Inevitable toxicity must be accepted and minimised by alteration in treatment providing cure is not jeopardised. Lifetime follow-up is essential for all these patients but they themselves are the best monitors of their health and they must be guided to accept responsibility and to be the vanguard of educators of the profession and the public. They must not feel the cost of cure has been too great.

**References**

ABRAMSON, D.H., RONNER, H.J. & ELLSWORTH, R.M. (1979). Second tumours in non-irradiated bilateral retinoblastoma. *Am. J. Ophthalmol.*, **87**, 624.

ARNEIL, G.C., EMMANUEL, I.G., FLATMAN, G.E., HARRIS, F. YOUNG, D.G. & ZACHARY, R.B. (1974). Nephrulism two children after irradiation and chemotherapy for nephroblastoma. *Lancet*, **i**, 960.

BIRCH, J.M., MARSDEN, H.B., MORRIS JONES, P.H., PEARSON, D. & BLAIR, V. (1988). Improvements in survival from childhood cancer in results of a population based study over 30 yrs. *Br. Med. J.*, **296**, 1372.

EISER, C. (1991). Cognitive deficits in children treated for leukaemia. *Arch. Dis. Child.*, **66**, 164.

GREEN, D.M. (1989). Long term complications of therapy for cancer in childhood & adolescence. The Johns Hopkins University Press: Baltimore, Maryland.

GREEN, D.M., GUNGELL, R.L., PEARCE, J., PANOHON, A.M. & GHOORAH, J. (1987). The effecet of mediastinal radiation on cardiac function of patients treated during childhood and adolescence for Hodgkin's Disease. *J. Clin. Oncol.*, **5**, 239.

HAWKINS, M.M., DRAPER, G.J. & KINGSTON, J.E. (1987). Incidence of second primary tumours among childhood cancer survivors. *Br. J. Cancer*, **56**, 339.

HAWKINS, M.M., KINGSTON, J.E. & KINNIER-WILSON, L.M. (1990). Late deaths following treatment for childhood cancer. *Arch. Dis. Child.*, **65**, 1356.

HEYN, R., RAGAB, A., RANEY, R.B. & 5 others (1986). Late effects of therapy in orbital rhabdomyosarcoma in children. *Cancer*, **57**, 1738.

LEGHA, S.S., BENJAMIN, R.S., MACKAY, B. & 6 others (1982). Reduction of doxorubicin cardiotoxicity by prolonged continuous intravenous infusion. *Ann. Int. Med.*, **96**, 133.

LIBSCHITZ, H.I. & EDEIKIN, B.S. (1981). Radiotherapy changes of the paediatric hip. *Am. J. Roentgenol.*, **137**, 585.

LI, F.P., CASSADY, J.R. & JAFFE, N. (1975). Risk of second tumours in survivors of childhood cancer. *Cancer*, **35**, 1230.

MEADOWS, A.T., BAUM, E., FOSSATI BELLANI, F. & 10 others (1985). Second malignant neoplasms in children. An update of the late effects study Group. *J. Clin. Oncol.*, **3**, 532.

MILLER, D.R. (1988). Late effects of childhood cancer. *Am. J. Dis. Child.*, **142**, 1147.

MOULD, J.J. & ADAM, N.M. (1983). The problem of avascular necrosis of bone in patients treated for Hodgkin's Disease. *Clin. Radiol.*, **34**, 231.

PECKHAM, V.C., MEADOWS, A.T., BARTEL, N. & MARRERO, O. (1988). Educational late effects in long term survivors of childhood acute lymphoblastic leukaemia. *Pediatrics*, **81**, 127.

PROBERT, J.C., PARKER, B.R. & KAPLAN, H.S. (1973). Growth retardation after megavoltage irradiation of the spine. *Cancer*, **32**, 634.

QUIGLEY, C., COWELL, C., JUMINEZ, M. & 6 others (1989). Normal or early development of puberty despite gonadal damage in children treated for acute lymphoblastic leukaemia. *N. Engl. J. Med.*, **321**, 143.

RUBIN, P., ANDREWS, J.R., SWARM, R. & GRUMP, H. (1959). Radiation induced dysplasias of bone. *Am. J. Roentgenol.*, **89**, 206.

SEXAUER, C.L., KHAN, A., BURGER, P.C. & 4 others (1985). Cisplatin in recurrent brain tumours. *Cancer*, **56**, 1497.

SHALET, S.M. (1989). Endocrine consequences of the treatment of malignant disease. *Arch. Dis. Child.*, **64**, 1635.

STEINHERTZ, L.J., STEINHERZ, P. & TAN, C. (1987). Cardiac failure more than six years post anthracyclines. *Proceedings of the XIXth Meeting of the International Paediatric Oncology Society, Jerusalem.* 136.

STILLER, C.A. & BUNCH, K.J. (1990). Trends in survival of childhood cancer in Britain diagnosed 1971–85. *Br. J. Cancer*, **62**, 806.

TORTI, F.M., BRISTOW, M.R., HOWES, A.E. & 6 others (1983). Reduced cardiotoxicity of doxorubicin delivered on a weekly schedule. *Ann. Intern. Med.*, **99**, 745.

WALLACE, W.H.B., SHALET, S.M., HENDRY, J.H., MORRIS JONES, P.H. & GATTAMANENI, H.R. (1989). Ovarian failure following abdominal irradiation in childhood. The radio-sensitivity of the human oocyte. *Br. J. Radiol.*, **62**, 995.